# Deposition of CENP-A^Cse4^ is enhanced by mutations in the AAA^+^ ATPase domain of ATAD2^Yta7^

**DOI:** 10.1093/genetics/iyag035

**Published:** 2026-02-05

**Authors:** Navpreet Kaur, Carol Cho, Anke Samel-Pommerencke, Sara Shahnejat-Bushehri, Alexandra Poßling, Jolita Kuznecova, Ji-Joon Song, Ann E Ehrenhofer-Murray

**Affiliations:** Institut für Biologie, Humboldt-Universität zu Berlin, 10099 Berlin , Berlin, Germany; Department of Biological Sciences and KAIST Stem Cell Center, Korea Advanced Institute of Science and Technology (KAIST), Daejeon 34141, Daejeon, Korea; Institut für Biologie, Humboldt-Universität zu Berlin, 10099 Berlin , Berlin, Germany; Institut für Biologie, Humboldt-Universität zu Berlin, 10099 Berlin , Berlin, Germany; Institut für Biologie, Humboldt-Universität zu Berlin, 10099 Berlin , Berlin, Germany; Institut für Biologie, Humboldt-Universität zu Berlin, 10099 Berlin , Berlin, Germany; Department of Biological Sciences and KAIST Stem Cell Center, Korea Advanced Institute of Science and Technology (KAIST), Daejeon 34141, Daejeon, Korea; Institut für Biologie, Humboldt-Universität zu Berlin, 10099 Berlin , Berlin, Germany

**Keywords:** centromere, kinetochore, Cbf1, Ubr2, Mub1

## Abstract

The chromatin remodeling factor and histone chaperone Yta7 is a member of the ATAD2 family of AAA^+^ ATPases from *Saccharomyces cerevisiae* that has *in vivo* functions consistent with both nucleosome assembly and disassembly activity. At the centromere, Yta7 is required for proper deposition of the centromeric histone H3 variant CENP-A^Cse4^. Here, we performed a genetic screen to identify suppressors of the defect of a mutation in CENP-A^Cse4^ that impairs the interaction with the DNA of the centromeric nucleosome (*cse4-S135A*). This identified two suppressor alleles of *YTA7*, *yta7-R483S and -D518E*, which are in the AAA1 domain of Yta7. Interestingly, Yta7-R483S enhanced the deposition of CENP-A^Cse4^ at the centromere and showed a ∼40% increased ATPase activity, suggesting that the hyperactivity of the motor domain is responsible for suppression of the *cse4-S135A* growth defect. In contrast, Yta7-D518E showed reduced ATPase activity, but both Yta7-R483S and -D518E retained the interaction with CENP-A^Cse4^ and centromeric sequences as well as hexamer formation *in vitro*. Our analysis of *in vivo* interactions between Yta7 and CENP-A^Cse4^ further showed that the two AAA^+^ domains and the non-canonical bromodomain of Yta7 are necessary and sufficient for interaction with CENP-A^Cse4^. The genetic screen furthermore revealed a mutation in the chromatin remodeler Fun30 as a suppressor of the centromeric defect of *cse4-S135A*. Altogether, this work reveals unusual, hypermorphic properties of Yta7 variants and highlights the importance of nucleosome remodelers in establishing centromeric chromatin.

## Introduction

Centromeres are specialized regions of eukaryotic chromosomes that serve as the primary sites for microtubule attachment, thus enabling accurate chromosome segregation during mitosis and meiosis. In higher eukaryotes, centromeric DNA consists largely of α-satellite repeats that are packaged into a distinct type of chromatin. This chromatin is characterized by the presence of both canonical nucleosomes containing histone H3, and nucleosomes with the histone H3 variant CENP-A (Musacchio and Desai 2017). To assemble the CENP-A containing nucleosomes, eukaryotic cells use the CENP-A specific histone chaperone, HJURP (Dunleavy et al. 2009; Foltz et al. 2009), which is recruited to the centromere by the Mis18 complex (Fujita et al. 2007). The presence of CENP-A nucleosomes is critical, as they mark chromatin as centromeric and recruit the inner kinetochore protein complex known as the constitutive centromere-associated network (CCAN) complex (Hori et al. 2008; Pesenti et al. 2022; Yatskevich et al. 2023). CCAN, in turn, connects to three subcomplexes of the outer kinetochore, collectively termed the KMN network (KNL1^SPC105^, MIS12^MIND^, and NDC80), the microtubule-binding entity being the NDC80 complex (Dimitrova et al. 2016). Altogether, these complexes form the kinetochore that provides a physical connection between chromatin and the spindle microtubules, thus ensuring proper chromosome alignment and segregation.

In metazoans, centromeres consist of a broad zone of H3- and CENP-A-containing nucleosomes (“regional” centromeres). In contrast, centromeres in the yeast *Saccharomyces cerevisiae* carry a single CENP-A containing nucleosome (a “point centromere”) that occupies defined centromeric DNA sequences on the individual chromosomes (termed CEN, consisting of the DNA elements CDEI, CDEII and CDEIII) (Musacchio and Desai 2017). The CENP-A homolog, which is termed Cse4 in *S. cerevisiae*, is homologous to CENP-A in the core region of the histone, but in addition has an extended *N*-terminal domain (Cse4N) of 135 amino acids, a feature that is only found in closely related yeasts, but not in higher eukaryotes (Stoler et al. 1995; Meluh et al. 1998). Despite this difference, the protein components and subcomplexes of *S. cerevisiae* kinetochores are remarkably similar to those of higher eukaryotes, and many of the kinetochore proteins were first identified in *S. cerevisiae*.

A key question in chromosome biology is how centromeres and kinetochores are regulated. To gain new insights into this process, we have previously employed yeast genetics to identify novel regulators of kinetochore function. Three of these studies are particularly relevant to the present work. In one study, we identified two posttranslational modifications (PTMs) in Cse4N, the methylation of arginine 37 (Samel et al. 2012) and the acetylation of lysine 49. Mutation of the respective amino acid positions caused a kinetochore defect when the centromere was compromised by mutations or deletions in genes encoding components of the CCAN complex and with the CDEI-binding protein Cbf1. Moreover, using a genetic screen for suppressors of this defect, we found mutations in two components of the CCAN complex, CENP-Q^Okp1^/CENP-U^Ame1^, to suppress this defect. Further experiments showed that CENP-Q^Okp1^/CENP-U^Ame1^ physically interact with Cse4N, that the suppressing alleles enhance the affinity to Cse4N, and that the interaction is inhibited by the two PTMs in Cse4N (Anedchenko et al. 2019).

In the same genetic suppressor screen (Anedchenko et al. 2019), we also found that the deletion of the genes encoding the E3 ubiquitin ligase Ubr2 and its adaptor protein Mub1 suppress the defect caused by the absence of Cse4-R37 methylation (*cse4-R37A*). Ubiquitination by Ubr2 is known to cause proteasomal degradation of the Dsn1 protein (Akiyoshi et al. 2013), which is a component of the MIND complex, and accordingly, overexpression of *DSN1* also suppressed the defect of *cse4-R37A* (Samel et al. 2017).

In another screen to uncover novel regulators of centromere function, we isolated suppressor mutations of the growth defect caused by mutation of Cse4-R37 (*cse4-R37A*) combined with *okp1-5*, an allele of the essential *OKP1* gene. This screen revealed mutations in *YTA7* and *UBR2* as suppressors. As will be discussed below, Yta7 is a histone chaperone and chromatin remodeler. We further found that the deletion of *YTA7* (*yta7Δ*) causes strong centromeric defects in a broad range of kinetochore mutants, which is accompanied by a decrease of Cse4 levels at the centromere in the absence of Yta7. Yta7 interacts with Cse4 in the cell, as determined by co-immunoprecipitation, and Yta7 is associated with centromeres. Altogether, we concluded that Yta7 cooperates with the CENP-A chaperone Scm3 (the functional equivalent of HJURP (Camahort et al. 2007; Stoler et al. 2007)) to deposit Cse4 at the centromeres (Shahnejat-Bushehri and Ehrenhofer-Murray 2020).

**Fig. 1.**
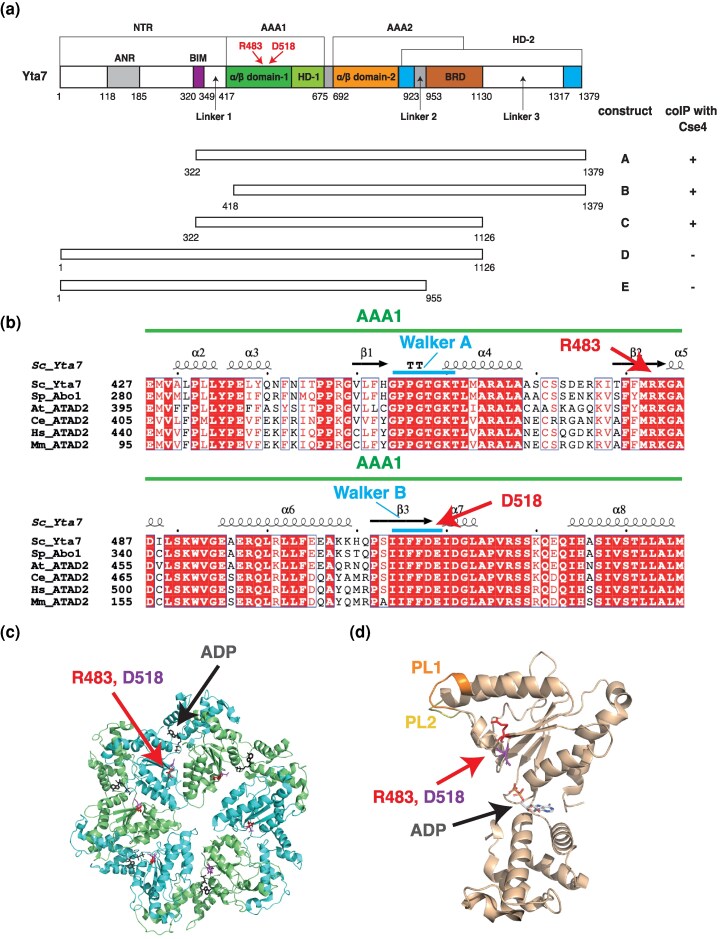
Domain architecture of Yta7. a) Location of domains in Yta7 (adapted from (Wang et al. 2023)). ANR, acidic N-terminal region; BRD, bromodomain; BIM, BRD-interacting motif; AAA1 and AAA2, AAA^+^ ATPase domains 1 and 2; HD, helical domain. Numbers below the bar show amino acid positions. White regions were not resolved in the cryo-EM structure of Yta7 (Wang et al. 2023). The Yta7 constructs A–E tested in this study for co-immunoprecipitation (co-IP) with Cse4 and the outcome are shown below (+, co-IP between Yta7 and Cse4 observed; -, no co-IP) (see Fig. 5, Supplementary Fig. 4). b) Multiple sequence alignment of the AAA1 domain of YTA7 and homologs showing the Walker A and Walker B motifs and the location of *yta7* suppressor alleles characterized in this study (*yta7-R483S* and *-D518E*). The location of α-helices and β-strands in *S. cerevisiae*  Yta7 is given above the sequences. The Yta7 homologs are: Abo1 (*Schizosaccharomyces pombe*) and ATAD2 from *Arabidopsis thaliana* (At_ATAD2), *Caenorhabditis elegans* (Ce_ATAD2), *Homo sapiens* (Hs_ATAD2) and *Mus musculus* (Mm_ATAD2). Sequences were aligned using the Clustal W program and displayed using ESPRIPT3.0 (Robert and Gouet 2014). The conserved domains are labeled. Red represents 100% identity among sequences (Madeira et al. 2024). c) and d) Location of Yta7-R483 and *-*D518 in the Cryo-EM structure. c) shows the Yta7 hexamer with the monomers colored in blue and green and the position of the respective residues. d) shows the Yta7 monomer (ADP-bound form, PDB 7UQK) (Wang et al. 2023).

Yta7 is a chromatin remodeling factor and histone chaperone involved in transcription and DNA replication. It is molecularly distinct from the classical chromatin remodelers like the SWI/SNF, ISWI or INO80 complexes, since it contains two tandem AAA^+^ ATPase domains (AAA1 and AAA2), a feature not found in the other remodelers, and only AAA1 has catalytic activity (Wang et al. 2023). Furthermore, Yta7 carries a non-canonical bromodomain (Fig. 1a) (Gradolatto et al. 2009). As a type II AAA^+^ ATPase, Yta7 bears similarity to so-called molecular segregases like NSF or p97/Cdc48, in which the AAA^+^ monomers oligomerize into hexameric rings with ATP-binding pockets that are formed at the interface between subunits (Puchades et al. 2020). Such hexamers use the energy from ATP hydrolysis to disassemble protein complexes, aggregates and diverse other polymers by binding to substrates and unfolding them by translocating them through the central pore of the hexameric ring (Puchades et al. 2020). Similar to these homologs, Yta7 monomers assemble into hexameric rings. Since Yta7 has two AAA^+^ domains, it forms two stacked rings with its AAA1 and AAA2 domains, and a third stack is formed by a hexamer of the bromodomains and lies on top of AAA1 (Wang et al. 2023). In agreement with its similarity to unfoldases of the AAA^+^ family of proteins, Yta7 can disassemble chromatin *in vitro* to release H3/H4, and its activity stimulates DNA replication in an *in vitro* chromatin replication assay. Yta7 therefore has been termed a chromatin segregase (Chacin et al. 2021). Of note, the disassembly activity as well as the *in vitro* ATPase activity of Yta7 require its phosphorylation by the S-phase Cyclin-Dependent Kinase (S-CDK). So far, seven S-CDK dependent phosphorylation sites have been identified in Yta7, which are clustered in its N-terminus and near the AAA1 domain (threonine (T) 67, serine (S) 94, T212, S259, S304, S380, and T445 (Kurat et al. 2011)). Yta7 is phosphorylated in the cell during S-phase, which is required for its cellular function during DNA replication. Interestingly, the non-canonical bromodomain of Yta7 binds histones independently of histone lysine acetylation, a feature that distinguishes it from other bromodomains (Gradolatto et al. 2009). However, recruitment of Yta7 to chromatin *in vitro* requires acetylation on histone H3 (Chacin et al. 2021).

The role of Yta7 in disassembling chromatin to allow DNA replication is in good agreement with its role in transcription. It localizes to the 5′ end of approx. 600 genes in the yeast genome and helps evict histones during gene induction (Lombardi et al. 2011). Conversely, Yta7 has been described as a repressor at the histone gene locus, where its phosphorylation leads to eviction from the promoter and subsequent gene induction (Fillingham et al. 2009; Kurat et al. 2011; Zunder and Rine 2012). In the context of yeast centromeres, Yta7 formally classifies as a chromatin assembly factor, because *yta7Δ* causes a loss of Cse4 at CEN (Shahnejat-Bushehri and Ehrenhofer-Murray 2020). One hypothesis to reconcile these observations with its disassembly activity is that Yta7 interacts with and unfolds Cse4, perhaps in the context of a Cse4/H4 dimer or tetramer, and hands Cse4 to the dedicated CENP-A chaperone Scm3 for incorporation into centromeric chromatin.

Yta7 shares the hexamer structure with its homolog in *Schizosaccharomyces pombe,* termed Abo1 (Gal et al. 2016; Cho et al. 2019), as well as with the human homolog ATAD2 (ATPase family AAA domain-containing 2) (Cho et al. 2023). Interestingly, Abo1 is a global transcriptional repressor and chromatin assembly factor in *S. pombe*, thus illustrating the dual function of Yta7/ATAD2 homologs variably as chromatin disassembly and assembly factors. ATAD2 is a transcriptional regulator that was originally identified as a co-activator for several cancer-related transcription factors (Zou et al. 2009), but likely serves both as an activator and a repressor in a locus-specific manner (Morozumi et al. 2016). ATAD2 expression is upregulated in various cancers and correlates with unfavorable patient prognosis (Liu et al. 2022). Structural analysis of ATAD2-histone H3/H4 complex shows binding of the histones to the AAA1 ring, with the H3 N-terminus inserted into the central pore of the ring. Of note, a well-defined structure of ATAD2 could only be obtained when using a truncated version carrying a mutation in the Walker B motif (ATAD2- E532Q) that blocks ATP hydrolysis (Cho et al. 2023). Whether ATAD2, like Yta7, has a function at centromeres, has not been investigated in detail. However, ATAD2 was identified in a study to map CENP-A-associated proteins, suggesting that it may serve as a CENP-A chaperone (Remnant et al. 2019). Furthermore, expression of a chimeric version of the centromeric histone variant carrying the human CENP-A histone-fold domain fused to Cse4N in *S. cerevisiae* was detrimental in *yta7Δ*, but not wild-type yeast, lending support to the notion that CENP-A deposition is regulated by Yta7/ATAD2 (Olafsson et al. 2023).

In this study, we sought to identify novel regulators of centromere function in *S. cerevisiae*. Using a genetic screen, we isolated mutations that suppress the defect caused by mutation of serine 135 (S135A) in Cse4. This site lies close to the DNA that is wrapped on the CENP-A^Cse4^ nucleosome in the structure of yeast inner kinetochore (Yan et al. 2019; Dendooven et al. 2023), suggesting that the mutation reduces nucleosome stability (Supplementary Fig. 1). Interestingly, we found that two point mutations in Yta7, -R483S and -D518E, but not the *YTA7* deletion, suppress the defect. Of note, the mutated residues both are located in AAA1, and D518E lies in the Walker B motif. Yta7-R483S showed ∼40% increased ATPase activity *in vitro*, suggesting that hyperactivity of the motor domain, and thus possibly unfolding activity, stimulates CENP-A^Cse4^ deposition at the centromere. Conversely, the activity of Yta7-D518E was reduced, and both variants retained interaction with Cse4, the ability to bind centromeric sequences, and the ability to form hexamers *in vitro*, indicating that D518E acts by a distinct mechanism. Further, we found that a minimal Yta7 fragment containing AAA1, AAA2, BRD and BIM, is sufficient for interaction with Cse4 in yeast cells.

Besides the mutations in Yta7, we also found mutations in *UBR2, MUB1* and *FUN30* as suppressors of *cse4-S135A*. Fun30 is a SWI/SNF-like chromatin remodeler (Awad et al. 2010; Durand-Dubief et al. 2012), thus reinforcing the notion that chromatin remodeling is required for proper centromere function. Furthermore, the absence of Ubr2/Mub1 increases the levels of the MIND component Dsn1, which explains this suppression (Samel et al. 2017). Altogether, these findings highlight how multiple pathways cooperate to maintain centromeric chromatin.

## Material and methods

### Yeast strains and plasmids

The yeast strains and plasmids used in this study are listed in Supplementary Tables 1 and 2. Yeast was grown and manipulated according to standard procedures (Sherman 1991). Yeast was grown on full medium (YPD) and selective minimal plates (YM), and plates containing 5-fluoro-orotic acid (5-FOA) (US Biological) were used to select against *URA3*.

Genomic integrations of *yta7-R483S* and *yta7-D518E* were constructed as follows: The alleles were generated by site-directed mutagenesis and cloning into pRS306-*YTA7* plasmids, which were subsequently linearized in *YTA7* using NheI and used to transform AEY1 to uracil prototrophy. Transformants were recovered and restreaked on 5-FOA medium to select for loss of *URA3*. 5-FOA-resistant colonies were tested for the presence of the *yta7* allele by PCR-amplification and sequencing.

C-terminal truncations and epitope-tagging of Yta7 were generated by genomic integration of the 9xmyc epitope tag. The site of the Yta7 truncation was determined by the primers used for PCR amplification of the epitope tag and the marker gene (Supplementary Tables 3 and 4). N-terminal truncations were obtained by integrating the *GALS* promotor 5′ to *YTA7* using pYM-N13 (Janke et al. 2004). The size of the truncation was determined by the PCR primers used for amplification of the promotor and marker gene (Supplementary Tables 3 and 4). All genomic integrations were verified by PCR amplification, and successful epitope tagging was verified by Western blotting.

Strains carrying 9xmyc-tagged *YTA7* alleles (*yta7-R483S, yta7-D518E*) and 3xHA-*CSE4* or 3xHA-*cse4-S135A* were generated by genomic integration of the 9xmyc tag C-terminal to *YTA7* in *yta7-R483S* or *yta7-D518E* strains, followed by genetic crosses and plasmid shuffle to introduce *cse4Δ* and plasmids pRS426-3xHA-*CSE4* or pRS423-3xHA-cse4-S135A. Epitope tags were verified by Western blotting.

### Screen for suppressors *cbf1Δ cse4-T133A-S135A-E136A* and whole-genome sequencing

The *cbf1Δ cse4Δ* + p*URA3*-*cse4-T133A-S135A-E136A* strain (AEY6629) was spread at a density of 3 × 10^6^ cells on YPD plates. For UV mutagenesis, plates were UV-irradiated with 3,000 µJ/cm^2^ (wavelength 254 nm) in a Stratalinker 2400 UV Crosslinker and subsequently incubated for four to five days at 37 °C in the dark. This UV dosage gives a survival rate of approx. 60% when cells are grown at 23 °C. In a separate approach, spontaneous revertants were isolated. Surviving colonies were restreaked and grown on YPD at 23 °C. Growth at different temperatures was determined by plating serial dilutions of the strains on YPD and incubating the plates at different temperatures for three days.

To test for dependence of the growth phenotype on the *cse4-T133A-S135A-E136A* allele, temperature-resistant candidates were transformed with a *HIS3*-marked *cse4-T133A-S135A-E136A* plasmid, p*URA3*-*cse4-T133A-S135A-E136A* was eliminated by counterselection on 5-FOA medium, and the resultant strain was tested for temperature resistance.

Six candidates that retained temperature resistance after plasmid shuffle (three spontaneous, three UV-induced) were backcrossed to a *cse4Δ* p*URA3-CSE4* strain (AEY5688), tetrads dissected and *cbf1Δ cse4Δ* p*HIS3*-*cse4-T133A-S135A-E136A* segregants tested for temperature resistance.

For whole-genome sequence analysis (WGS), strains were grown individually, and groups of temperature-sensitive and temperature-resistant segregants from the backcrosses were mixed in equal quantities of cells (OD_600_ equivalents). DNA was extracted from 12 such pools (six pools of temperature-resistant, and six pools of temperature-sensitive segregants), sequencing libraries were prepared, and sequencing was conducted on an Illumina NovaSeq6000 instrument (paired-end sequencing, 150 bp) to obtain approx. 5 × 10^6^ read pairs per pool. Reads were aligned to the reference genome (SacCer3) using Bowtie2, and single-nucleotide polymorphisms (SNPs) were called using deepSNV by comparing aligned reads from the pools of temperature-resistant to temperature-sensitive segregants from a given backcross (Gerstung et al. 2012).

### Yeast protein extracts, co-immunoprecipitation, and Western blotting

For Western blot analysis, 8 OD of cells were harvested, washed once with TBS and resuspended in 100 μL lysis buffer (1 × PBS containing 0.1% NP-40, 1 mM EDTA and protease inhibitor). Cells were lysed by bead beating (using a FastPrep- 5G Homogenizer MP-Biomedical) for 45 s at the homogenizing intensity. Loading buffer was added to each sample, and samples were heated for 5 min to 95 °C. Protein extracts equivalent to 1 OD_600_ of cells were analyzed by Western blot. Antibodies used for Western blotting were α-HA (Covance MMS-101P), α-c-Myc antibody (9E10, Invitrogen), α-H2B (Active Motif 39237) and α-H2A (Active Motif 39235).

For co-immunoprecipitation, yeast strains were grown at 23 °C. 200 OD_600_ yeast cells were harvested and lysed by bead-beating in 1 mL of cold IP lysis buffer (50 mM HEPES, 200 mM sodium acetate, 0.25% Nonidet P-40, 1 mM EDTA, 1 mM EGTA, 5 mM magnesium acetate, 5% glycerol, 3 mM DTT, 1 mM PMSF and protease inhibitors). The whole-cell lysate was cleared by centrifugation, and samples were normalized for their protein concentration before being used for the IP. An aliquot of 100 μL was taken as input control. 600 μL of each sample was incubated with 5μL of α-Myc antibody overnight followed by 2 h incubation with 50 µl of Protein G dynabeads at 4°C. For immunoprecipitation of HA-tagged Cse4 using α-HA agarose, the resin was pre-washed 5 times with lysis buffer prior to overnight incubation with lysate. 70 μL of α -HA agarose (Sigma, A2095) was added to 600 μL samples. Protein-antibody-bead/agarose conjugates were washed 3 times with lysis buffer and suspended in 50 μL of sample loading buffer (final concentration 62.5 mM Tris pH 6.8, 2% SDS, 10% glycerol, 5% 2-mercaptoethanol, 0.001% bromophenol blue). α-Myc antibody was obtained from Thermo Fisher Scientific (MA1-980) and used at a 1:500 dilution for Western blotting. α-HA-antibody (Covance) was used at 1:250 or 1:500. The immunoblots for detection of Yta7 and Cse4 were obtained from the same gel and Western blot by cutting the blot at the 55 kD marker and incubating the upper part with α-Myc (detection of Yta7) and the lower part with α-HA (detection of Cse4) antibody. Blots were imaged on a Bio-Rad imaging system. Quantification of Western blots was performed with ImageJ software.

### Chromatin immunoprecipitation (ChIP)

Cells were grown to an OD_600_ of 0.6–0.9 at 23 °C. 30 OD_600_ cells were cross-linked with 1% formaldehyde for 10 min at room temperature. The cross-linking reaction was stopped by adding glycine to a final concentration of 125 mM. Cells were washed twice with TBS and once with spheroplasting buffer (1 M sorbitol, 100 mM KPO_4_ and 30 mM β-mercaptoethanol) and then resuspended in spheroplast buffer with 40 unit/mL zymolyase and incubated for 30 min at 30 °C. Spheroplasts were washed twice with spheroplast buffer and then resuspended in ice-cold lysis buffer (50 mM HEPES pH 7.5, 1 mM EDTA, 140 mM NaCl, 1% Triton X-100, 0.1% Na-Deoxycholate, 0.2 mM PMSF, 1 µg/mL leupeptin, 1 µg/mL pepstatin). The chromatin was sheered with a Bioruptor (Diagenode, 15 cycles 30 s on, 30 s off). To remove cell debris, the lysate was centrifuged and the supernatant retained. From fragmented chromatin samples, aliquots were taken as an input control for the quantitative real-time PCR and as a control for the average chromatin fragment size. The remaining chromatin samples were pre-cleared with Dynabeads protein G (Thermo Fisher Scientific) for 2 h at 4°C and incubated overnight with 4 μL of α-Myc, α-HA antibody (Covance) or α-histone H4 antibody (Abcam ab7311). Dynabeads Protein G was subsequently added, and samples were incubated for 2 h at 4 °C. The immunoprecipitates were recovered with a magnet and washed sequentially with 1 mL of the following buffers: (1) low salt solution (0.1% (v/v) SDS, 1% (v/v) Triton X-100, 2 mM EDTA, 20 mM Tris (pH 8.1) 150 mM NaCl); (2) high salt solution (0.1% (v/v) SDS, 1% Triton (v/v) X-100, 2 mM EDTA, 20 mM Tris (pH 8.1) 500 mM NaCl); (3) LiCl buffer (0.25 M LiCl, 1% (v/v) Nonidet P-40, 1% (w/v) sodium deoxycholate, 1 mM EDTA, 10 mM Tris pH 8.1), twice 1 × TE. The precipitated chromatin was resuspended in 100 µl 10% Chelex 100 resin (Bio-Rad) and boiled for 10 min to reverse cross-linking. Protein was digested with proteinase K, and samples were then RNase-treated, and IP DNA was obtained by collecting the supernatant after centrifugation. To extract the input DNA, the samples were incubated overnight at 65 °C to reverse cross-linking. They were then treated 1 h with proteinase K and 1 h with RNase following by extraction with 10% Chelex 100 resin. Samples were subsequently analyzed by quantitative real-time PCR.

### Yeast strain construction and growth for Yta7 purification

For overexpression of *YTA7* in *S.cerevisiae*, codon optimized Yta7 (Genscript) was cloned into the AscI and SwaI sites of a modified pRS303 vector (gift from Dr. Stephen P. Bell's lab) that encodes a bi-directional Gal1:10 promoter, a C-terminal TEV protease site, and a Flag tag. The R483S and D518E mutations were introduced by inverse PCR with KOD polymerase (Toyobo). The cloned Yta7 expression plasmid vectors were digested with NheI restriction enzyme, transformed into the yMH09 background strain (gift from Dr. Stephen P. Bell's lab, genotype *MATa ade2-1 trp1-1 leu2-3, 112 his3-11, 15 ura3-1 can1-100 bar1::HisG lys2::HisG pep4::unmarked*), and selected on medium lacking histidine for genomic integration.

Yeast was grown at 30 °C in YEP supplemented with 2% glycerol(v/v) to an OD_600_ of ∼1.0, and arrested in G1 with 0.150 ng/mL ɑ-factor (Genscript) for 2 h. Yta7 protein expression was induced by the addition of galactose to 2% (w/v) for 3.5 −5 h and harvested by centrifugation at 4,500 × g for 10 min. Harvested cells were washed with cold milliQ water, resuspended in approximately one-third of packed cell volume of yeast lysis buffer (25 mM HEPES pH 7.5, 200 mM NaCl, 5% glycerol) supplemented with 1 mM PMSF and 1 tablet of cOmplete ULTRA^™^ protease inhibitor cocktail (Roche), and frozen dropwise as pellets in liquid nitrogen.

### Yta7 protein purification from yeast

Frozen yeast pellets were ground in an electric coffee grinder and thawed in a water bath at room temperature. Lysate was clarified by centrifugation at 29,000 × g for 1.5 h and bound to anti-DYKDDDK G1 affinity resin (Genscript) for 2.5 h. Affinity resin was washed with 5 CV Wash I buffer (25 mM HEPES pH 7.5, 500 mM NaCl, 5% glycerol), followed by 10 CV Wash II buffer (25 mM HEPES pH 7.5, 1,000 mM NaCl, 5% glycerol), 5 CV Wash I buffer, and 5 CV of lysis buffer (25 mM HEPES pH 7.5, 200 mM NaCl, 5% glycerol). Protein was eluted by incubating resin with lysis buffer supplemented with 0.4 mg/mL Flag peptide (synthesized by Shanghai Apeptide), and fractionated 1 CV each. Peak fractions with high Yta7 expression (typically fractions 2 to 5) were pooled and concentrated with a 100 kDa MWCO Amicon Ultracentrifugal Filter (Millipore), and run over a Superose 6 increase 10/300GL column (Cytiva Life Sciences) in 25 mM HEPES, pH 7.5, 200 mM NaCl, 5% glycerol, and 1 mM DTT. Peak fractions corresponding to hexameric Yta7 were concentrated and flash frozen in liquid nitrogen until further use. Typical protein yields were ∼10–20 µg purified protein per L cells.

### Sf9 insect cell growth for recombinant Yta7 expression

For expression of recombinant *S. cerevisiae*  Yta7 in Sf9 insect cells, codon optimized full length Yta7 (Genscript) or truncated Yta7 (Δ aa1-393) was cloned into the EcoRI and XhoI sites of a modified pFastBac1 expression vector (Thermo Fisher Scientific) that encodes a C-terminal TEV protease site and a Flag tag. The R483S and D518E mutations were introduced by inverse PCR with KOD polymerase (Toyobo). Bacmids were prepared by transformation of DH10Bac competent cells and transfected into Sf9 cells to produce baculovirus as detailed by the Bac-to-Bac Baculovirus Expression Kit (Thermo Fisher Scientific). Baculovirus was amplified by two successive rounds of Sf9 cell supernatant collection and re-infection. Wild-type and mutant Yta7 proteins were expressed by infecting 1L Sf9 suspension cultures grown in CCM Media (GE Healthcare) to a concentration of 2.0 × 10^6^ cells/mL with 50 mL of the final amplified baculovirus for 44–46 h. Cells were harvested by centrifugation at 4,500 × g for 10 min and resuspended in 20 mL of Sf9 lysis buffer (50 mM Tris pH 8.0, 300 mM NaCl, 5% glycerol) supplemented with 1 mM PMSF and 1 tablet of cOmplete ULTRA^™^ protease inhibitor cocktail (Roche).

### Recombinant Yta7 protein purification from Sf9 cells

Frozen Sf9 cells were lysed by four cycles of freezing in liquid nitrogen and thawing in a water bath at room temperature. Lysate was clarified by centrifugation at 29,000 × g for 1.5 h and bound to anti-DYKDDDK G1 affinity resin (Genscript) for 2.5 h. Affinity column purification and elution was performed as with recombinant Yta7 purification from *S. cerevisiae*. Peak fractions with high Yta7 expression (typically fractions 2 to 5) were pooled, diluted to a NaCl concentration of 100 mM, and run over a HiTrapQ 5 mL column (Cytiva Life Sciences) with a 50 to 1,000 mM NaCl gradient. Peak fractions from the HiTrapQ column were concentrated with a 100 kDa MWCO Amicon Ultracentrifugal Filter (Millipore) and run over a Superose 6 increase 10/300GL column (Cytiva Life Sciences) in 25 mM HEPES, pH 7.5, 200 mM NaCl, 5% glycerol, and 1 mM DTT. Peak fractions corresponding to hexameric Yta7 were concentrated and flash frozen in liquid nitrogen until further use. Typical protein yields were ∼40–60 µg purified protein per L cells.

### Negative stain electron microscopy

Negative stain electron microscopy grids were prepared by applying a layer of in-house prepared carbon film onto 400 mesh high open area copper grids (Graticules Optics). Prior to protein application, grids were glow-discharged on a PELCO easiGlow glow discharge cleaning system (Ted Pella) with a current of 30 mA, a hold time of 20 s, and a wait time of 10 s. 3 µL of purified Yta7 diluted to a concentration of 0.01–0.03 mg/mL was applied to the carbon side of a glow discharged grid. After 1 min of incubation, excess protein solution was adsorbed with filter paper. The grids were then washed with water and incubated with 1.5% (w/v) uranyl acetate for 1 min. Excess uranyl acetate was adsorbed with filter paper and grids were air dried for an additional minute. Stained and dried grids were imaged by a 200 keV Tecnai F20 electron microscope (FEI) at KAIST Analysis center for Research Advancement (KARA) at a magnification of 29,000×.

### ATPase assays

ATP hydrolysis activity of recombinant Yta7 protein was measured with the EnzChek Phosphate assay kit (Thermo Fisher Scientific). Yta7 was diluted in assay buffer (50 mM Tris pH 8.0, 100 mM NaCl, 1 mM DTT) and mixed with MESG substrate and PNPase enzyme to a final concentration of 3–5 nM in 96 well plates. After pre-incubation of the mixture for 5 min at 30 °C, ATPase reactions were initiated by addition of 1 mM MgATP. The absorbance at 360 nm was monitored on a Spark multimode microplate reader (Tecan) at 30 °C for 10 min with 10 s intervals. ATPase rates were determined by fitting the slope of the linear range of absorbance increase and dividing by hexameric Yta7 protein concentration.

## Results

### Mutations in *UBR2, MUB1* and *FUN30* suppress the defect of *cse4-T133A-S135A-E136A*

In earlier work, we have used genetic suppressor screens to identify regulators of centromere function. Importantly for this study, we found that specific mutations in the Okp1^CENP-Q^/Ame1^CENP-U^ heterodimer (*okp1-R164C* and ame1-273* (i.e. a stop codon at position 273 of *AME1*)) suppressed defects of mutations in the extended N-terminal domain of Cse4, which has led us to identify them as interaction partners of the N-terminus of Cse4 (Anedchenko et al. 2019). Here, to find novel centromere regulators, we searched for mutations in *CSE4* that cause a centromeric defect, but are not suppressed by *okp1-R164C* and ame1-273*, which suggests that they are not the result of a loss of interaction with Okp1^CENP-Q^/Ame1^CENP-U^. By investigating a collection of alanine scan mutations in *CSE4*, we identified the allele *cse4-T133A-S135A-E136A*, which on its own has no defect, but shows a temperature-sensitive growth defect when the centromere-binding protein Cbf1 is absent (*cbf1Δ*). Importantly, the *cbf1Δ cse4-T133A-S135A-E136A* defect was not suppressed by ame1-273* or *okp1-R164C* (Supplementary Fig. 2a), which indicates that the defect of the *cse4-T133A-S135A-E136A* allele is not due to the loss of interaction with Okp1^CENP-Q^/Ame1^CENP-U^. This conclusion is consistent with the finding that S135 of Cse4 is located close to the DNA wrapped around the histone core of the centromeric nucleosome (Supplementary Fig. 1). In a structure of the inner kinetochore of yeast, the “foot” region of Okp1^CENP-Q^/Ame1^CENP-U^ entities (Dendooven et al. 2023) (also termed “head domain” (Deng et al. 2023)), which bind to an α-helix formed by Cse4 aa 34–46, are located on the same side of the nucleosome as Cse4-S135, indicating that the remainder of Cse4N lies between these sites (Supplementary Fig. 1a). Furthermore, in a structure of CCAN on the Cse4 nucleosome, the positions Cse4-K130 and -L137 are at the nucleosome surface, while the intervening residues are not visible in the structure (Supplementary Fig. 1, b and c) (Yan et al. 2019).

We furthermore observed previously that *yta7Δ* suppresses centromeric defects in some contexts (Shahnejat-Bushehri and Ehrenhofer-Murray 2020), and we therefore tested whether *yta7Δ* affected *cbf1Δ cse4-T133A-S135A-E136A*. However, no suppression was observed (Supplementary Fig. 2b), showing that the defect of *cbf1Δ cse4-T133A-S135A-E136A* was not suppressed by the absence of Yta7.

Given these findings, we surmised that the identification of extragenic suppressors of the *cbf1Δ cse4-T133A-S135A-E136A* growth defect would yield novel insights into centromere regulation. We therefore isolated extragenic suppressors of the *cbf1Δ cse4-T133A-S135A-E136A* temperature sensitivity and identified the causative mutation by whole-genome sequencing (WGS). Operationally, a *cbf1Δ cse4-T133A-S135A-E136A* strain was plated at the restrictive temperature (37 °C), and temperature-resistant derivatives were isolated (Supplementary Fig. 2c, see Materials and Methods, either spontaneous revertants, or after UV mutagenesis). Six temperature-resistant derivatives were subsequently backcrossed to a *CBF1  CSE4* strain to eliminate non-causative background mutations. For each mutant, multiple temperature-resistant and temperature-sensitive *cbf1Δ cse4-T133A-S135A-E136A* segregants from the backcross were pooled, respectively, and subjected to WGS, the assumption being that a causative mutation should be present in all the temperature-resistant, but not in the temperature-sensitive segregants of the backcross. Using this approach, we were able to identify the causative mutation of five of the six revertants. For one revertant, we were unable to assign a causative mutation.

Interestingly, two *cbf1Δ cse4-T133A-S135A-E136A* revertants carry mutations in *UBR2* (stop codon at position 926) and *MUB1* (*mub1-Y479S*) (Supplementary Fig. 2c). *UBR2* encodes an E3 ubiquitin ligase, and *MUB1* encodes a Ubr2 adaptor protein (Akiyoshi et al. 2013). We have shown in earlier work that *ubr2Δ* and *mub1Δ* suppress centromeric defects caused by *cse4-R37A* mutation by stabilizing the outer kinetochore protein Dsn1 (Samel et al. 2017). The identification of the two suppressor mutations here was consistent with the earlier work and therefore was not further pursued.

A further temperature-resistant *cbf1Δ cse4-T133A-S135A-E136A* isolate was found to carry a mutation in *FUN30* (*fun30-M385R*, Supplementary Fig. 2c). Fun30 encodes a nucleosome remodeling factor with a function in DNA damage response and transcriptional silencing (Neves-Costa et al. 2009; Chen et al. 2012), and it is required for nucleosome positioning at and around the centromere (Durand-Dubief et al. 2012). The finding here of a *fun30* mutation as a suppressor of a centromeric defect is consistent with this earlier work and therefore was not further pursued here.

### The alleles *yta7-R483S* and *-D518E* suppress the defect of *cbf1Δ cse4-T133A-S135A-E136A*

Interestingly, we found that two temperature-resistant *cbf1Δ cse4-T133A-S135A-E136A* revertants carry mutations in *YTA7*, namely *yta7-R483S* and *-D518E*. This was unexpected, because our analysis above had shown that *yta7Δ* does not suppress the temperature-sensitive growth defect of *cbf1Δ cse4-T133A-S135A-E136A* (Supplementary Fig. 2b). This suggested that the two alleles do not constitute simple loss-of-function alleles, but rather may constitute hypermorphic alleles, and we therefore investigated them in detail in this study.

**Fig. 2.**
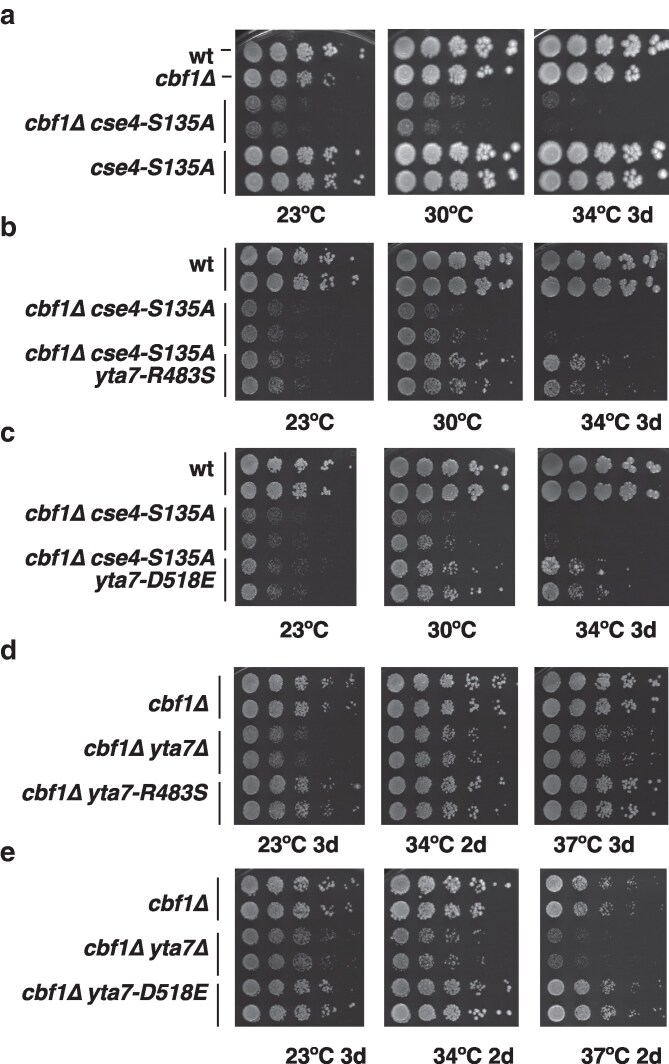
Mutation of serine 135 in CENP-A^Cse4^ (*cse4-S135A*) enhances the centromeric defect of *cbf1Δ* and is suppressed by *yta7-R483S* and *-D518E*. a) *cse4-S135A* enhances the growth defect of *cbf1*Δ. Serial dilutions of strains with the indicated genotypes were spotted on full medium and incubated for three days at the different temperatures. b) *yta7-R483S* suppressed the temperature-sensitive growth defect of *cbf1Δ cse4-S135A*. Representation as in a. c) *yta7-D518E* suppressed the temperature-sensitive growth defect of *cbf1Δ cse4-S135A*. d) and e), *yta7Δ*, but not *yta7-R483S* and *-D518E*, causes a defect in *cbf1Δ,* showing that they are not null alleles. For growth of *yta7-R483S* and *-D518E* alone, see Supplementary Fig. 2e.

Since *cse4-T133A-S135A-E136A* carries three amino-acid changes, we asked whether one of the amino-acid mutations alone was sufficient to cause a defect in *cbf1Δ*, and whether this defect was suppressed by the new *yta7* alleles. Indeed, *cse4-S135A* alone, but not the other two amino-acid changes alone, caused a temperature-sensitive growth defect in *cbf1Δ* (Fig. 2a, Supplementary Fig. 2d).

**Fig. 3.**
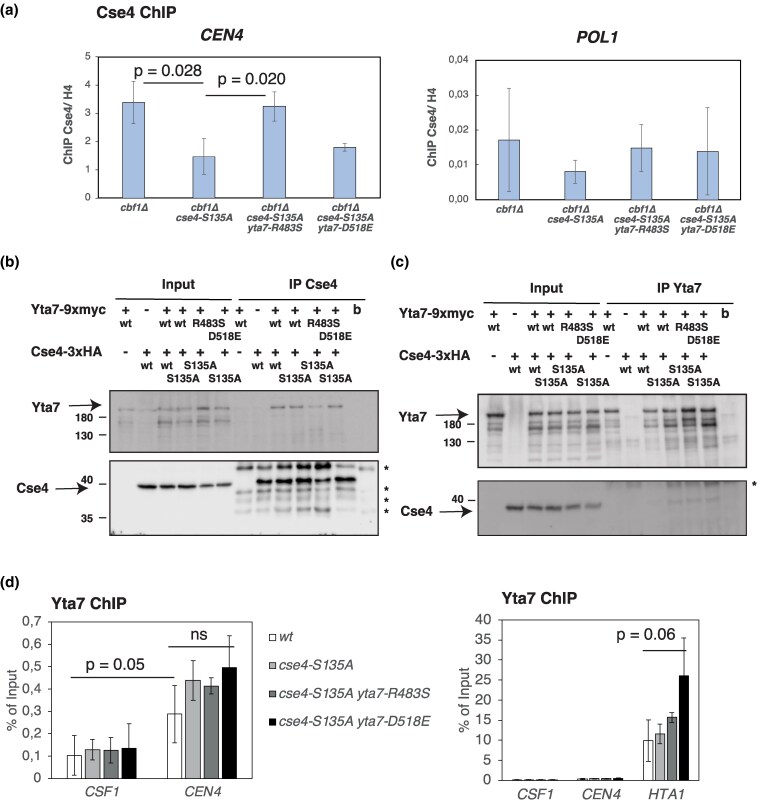
Yta7-R483S enhances the association of CENP-A^Cse4^ with the centromere. a) ChIP of CENP-A^Cse4^ at *CEN4*. Cse4-S135A is less strongly associated with *CEN4* than wild-type Cse4, and association is increased in *yta7-R483S*. Cse4 is not bound at the control site *POL1*. Mean values of biological triplicates and standard deviation are shown. *P*-values: Unpaired two-sided t-test. b) Immunoprecipitation (IP) of Cse4 or Cse4-S135A co-IPs Yta7, Yta7-R483S and -D518E. The top and bottom are a Western blot of the same gel. The upper part of the blot was incubated with α-Myc antibody to detect Yta7-9xmyc. The bottom part was incubated with α-HA to detect Cse4-3xHA. The asterisk (*) marks bands from the IP antibody that cross-react with the antibody used for Western blotting. Molecular weight markers (in kD) are shown on the left. c) as in b), but IP of Yta7 (b, beads only). Quantification of input and co-IP is shown in Supplementary Fig. 3b. d) Yta7-R483S and -D518E are associated with centromeric sequences. ChIP of Yta7 at *CEN4* and *CSF1* (as a control) are shown (left). Mean values of biological triplicates and standard deviations are shown. *P* value, unpaired two-sided t-test (comparison of Yta7 ChIP at *CEN4* compared to *CSF1*). Ns, no significant difference of Yta7 ChIP at *CEN4* in wt compared to *cse4-S135A yta7-D518E*. The enrichment of Yta7 at *CEN4* is statistically significant, but weaker than association with the promotor of *HTA1* (right).

To test the effect of *yta7-R483S* and *-D518E*, the alleles were introduced *de novo* into the *S. cerevisiae* genome to exclude potential effects of secondary mutations in the revertants. Importantly, both *yta7-R483S* and -*D518E* suppressed the temperature-sensitive growth defect of *cbf1Δ cse4-S135A*, the effect of -*D518E* being slightly weaker than that of -*R483S* (Fig. 2a–c), and neither allele alone caused a growth defect (Supplementary Fig. 2e). Of note, the alleles did not affect the level of Yta7 protein (see below, Fig. 3). This again supported the interpretation that these do not constitute null alleles of *YTA7*. To further corroborate this, we determined their effect in *cbf1Δ* alone (without *cse4-S135A*). We have shown earlier that *yta7Δ* shows a synthetic growth defect in *cbf1Δ*, which is due to reduced levels of Cse4 at the centromere in *cbf1Δ yta7Δ* (Shahnejat-Bushehri and Ehrenhofer-Murray 2020). Importantly, neither *yta7-R483S* nor *-D518E* caused a growth defect in *cbf1Δ* (Fig. 2, d and e), again arguing that they do not constitute null alleles.

### Yta7-R483S enhances Cse4 deposition at the centromere

We next sought to determine how Yta7-R483S and -D518E affect centromere function. Interestingly, both mutations lie in the N-terminal AAA^+^ domain of Yta7 (AAA1), and the residues are highly conserved across ATAD2 homologs (Fig. 1a). Yta7-R483 is located between the Walker A and Walker B motifs of AAA1, at the C-terminal end of a short β-sheet (β2), and D518 lies in the Walker B motif of AAA1 (Fig. 1b). In the three-dimensional structure of Yta7, the two sites are located close to each other at the interface between neighboring AAA1 domains in the Yta7 hexamer and close to the binding site for the ATP/ADP nucleotide (Fig. 1, c and d). To further interrogate these mutants, we tested (a) whether the Yta7 variants affect the deposition of Cse4 at the centromere, (b) whether they alter the interaction with Cse4 or with centromeric DNA, and (c) whether they affect the ATPase activity of Yta7.

To investigate the effect of the Yta7 variants on Cse4 deposition at the centromere, we determined the levels of wt Cse4 and Cse4-S135A relative to histone H4 in dependence of the *yta7* alleles by chromatin immunoprecipitation (ChIP). Significantly, the levels of Cse4-S135A were decreased at *CEN4* in *cbf1Δ* compared to wt Cse4 (Fig. 3a, left), suggesting that the growth defect of *cbf1Δ cse4-S135A* is due to insufficient amounts of Cse4 at the centromere. Of note, the total cellular levels of Cse4-S135A protein were not reduced compared to wt Cse4 (Supplementary Fig. 3a). Importantly, the levels of Cse4-S135A at *CEN4* were restored in *yta7-R483S*, showing that this *yta7* allele resulted in increased levels of Cse4-S135A at the centromere (Fig. 3a, left). For *yta7-D518E*, there was a tendency toward increased Cse4-S135A levels, but the effect was not statistically significant. This may explain why *yta7-D518E* suppresses the *cbf1Δ cse4-S135A* growth defect to a lesser extent (Fig. 2c). As a control, Cse4 and Cse4-S135A were not enriched at an unrelated gene, *POL1* (Fig. 3a, right, note the scale of the *y*-axis). Taken together, this showed that Yta7-R483S improves Yta7's ability to deposit Cse4 at the centromere.

We next asked whether the Yta7 variants show an altered interaction with Cse4 in the cell. To test this, we performed co-immunoprecipitation (co-IP) of Cse4 and Cse4-S135A with Yta7, Yta7-R483S and -D518E. The levels of cellular Cse4-S135A were not reduced compared to wt Cse4, and Yta7 levels were unaffected by the *yta7* alleles (Fig. 3b, input, see Supplementary Fig. 3b for quantification). Precipitation of Cse4 or -S135A showed co-IP of wild-type Yta7, indicating that Cse4-S135A did not have a reduced interaction with wild-type Yta7 (Fig. 3b, IP Cse4) (Shahnejat-Bushehri and Ehrenhofer-Murray 2020). Furthermore, both Yta7-R483S and -D518E could be co-IPed with Cse4-S135A, indicating that the Yta7 mutations did not affect the interaction between Yta7 and Cse4. Similarly, in the reverse immunoprecipitation (precipitating Yta7 and querying co-IP of Cse4, Fig. 3c), both Cse4 and Cse4-S135A were coIPed with Yta7 as well as with Yta7-R483S and -D518E (for quantification, see Supplementary Fig. 3b). These results showed that the *yta7* mutations did not affect the interaction between Yta7 and Cse4 in the cell.

We furthermore tested whether *yta7-R483S* and -*D518E* affected the ability of Yta7 to associate with centromeric sequences. However, chromatin immunoprecipitation (ChIP) showed that while all Yta7 variants were enriched at *CEN4* compared to an unrelated control locus, *CSF1* (Shahnejat-Bushehri and Ehrenhofer-Murray 2020), there were no statistically significant differences of Yta7-R483S or -D518E to wt Yta7 (Fig. 3d, left). As observed earlier (Shahnejat-Bushehri and Ehrenhofer-Murray 2020), the enrichment of Yta7 at *CEN4* was statistically significant, but was much lower than that at the promotor of the histone gene *HTA1* (Fig. 3d, right). At *HTA1*, Yta7-D518E showed a tendency toward stronger enrichment, but with low statistical confidence (*P* = 0.06, one-sided t-test).

### The mutation R483S enhanced the ATPase activity of Yta7

**Fig. 4.**
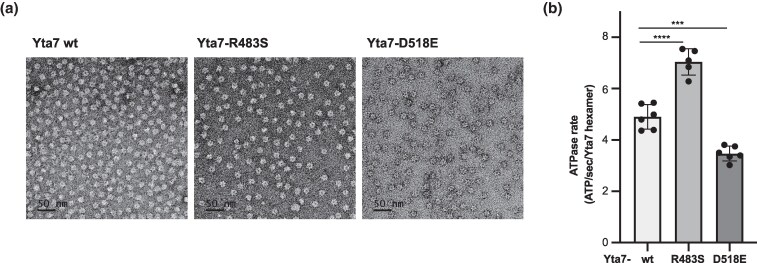
Yta7-R483S shows increased *in vitro* ATPase activity. a) Negative stain electron microscopy images of Yta7 variants purified from *S. cerevisiae* shows that the proteins form hexamers *in vitro*. Magnification 29,000×. b) Steady state ATPase rates of Yta7 proteins purified from *S. cerevisiae*. Mean values for Yta7 wt, -R483S, and -D518E are 4.9 ± 0.5, 7.0 ± 0.5, and 3.5 ± 0.3 ATP/sec/Yta7 hexamer, respectively. Error bars represent SD. ****P < 0.0001 and ***P < 0.001 (*n* = 6).

Since both Yta7-R483S and -D518E are in the AAA1 domain of Yta7, near the known catalytic site, we investigated whether these mutations affected Yta7's structural stability and catalytic activity. For this purpose, full-length Yta7 proteins harboring either suppressor mutation were overexpressed and purified from *S. cerevisiae* to high purity using affinity and size exclusion chromatography as previously described (Chacin et al. 2021; Wang et al. 2023). The wild-type and mutant Yta7 proteins showed no significant differences in protein purity, yield, or stability by SDS-PAGE (Supplementary Fig. 4a). Furthermore, both mutants also behaved similarly to wild-type on a native PAGE (Supplementary Fig. 4b) and displayed highly homogeneous particle distributions characteristic of the stable Yta7 hexamers by negative stain electron microscopy (EM) (Fig. 4a), indicating that the mutations did not disrupt Yta7 hexamer stability. Importantly, however, steady state ATPase assays revealed a statistically significant ∼40% increase in activity for Yta7-R483S compared to wild-type, suggesting a gain-of-function in ATPase activity (Fig. 4b). In contrast, Yta7-D518E displayed a ∼30% decrease in ATPase activity. Similar results were obtained from recombinant Yta7 proteins heterologously expressed in Sf9 insect cells, indicating that the mutations likely affect Yta7 directly (Supplementary Fig. 4a), rather than acting through interactions with other yeast proteins. The increased activity of Yta7-R483S was also observed in N-terminally truncated Yta7 proteins (Δ aa1-393, Supplementary Fig. 4b), which lack the primary Yta7 phosphorylation sites, demonstrating that the enhanced ATPase activity of Yta7-R483S results from altered catalytic function of the AAA1 motor domain, rather than from differential phosphorylation *in vivo*.

Taken together, the observation that Yta7-R483S is a hyperactive enzyme supports the notion that the increased ATPase activity enhances Cse4 deposition and thus suppresses the growth defect resulting from reduced Cse4-S135A levels at the centromere. In contrast, Yta7-D518E likely acts by a distinct mechanism (see discussion).

### Cse4 interacts with a fragment of Yta7 comprising AAA1, AAA2 and BRD

**Fig. 5.**
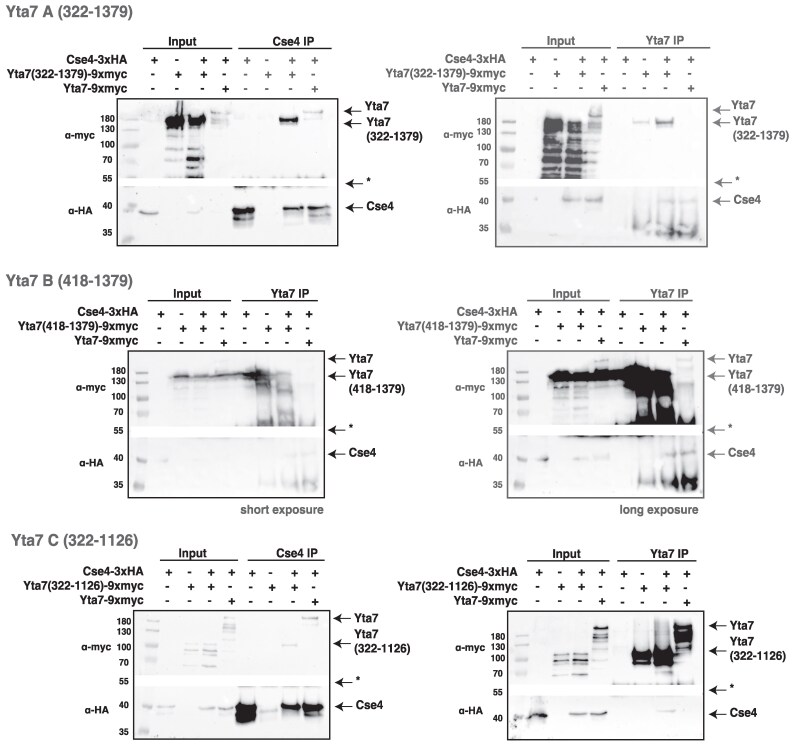
CENP-A^Cse4^ interacts *in vivo* with a minimal fragment of Yta7 (aa 322–1126) comprising AAA1, AAA2, BRD and BIM. The Yta7 constructs A (aa 322–1379, top), B (aa 418–1379, middle) and C (aa 322–1126, bottom) were epitope tagged with 9xmyc in yeast strains carrying Cse4-3xHA. Whole-cell extracts (input) were precipitated with Cse4 or Yta7, and the presence of Yta7 and Cse4 was determined by Western blotting, as shown in Fig. 3 (upper part of blot developed for Yta7 detection, lower part of the same blot for Cse4). The asterisk (*) indicates the place where the blot was cut (white box). For construct B (middle), a short (left) and a long (right) exposure of the same Western blot of precipitation with Yta7 are shown. Precipitation with Cse4 showed unspecific binding of construct B to the beads in the absence of tagged Cse4 (see Supplementary Fig. 5a). See Figure S5b and S5c for Yta7 constructs D and E.

Since the two *YTA7* suppressor alleles both lie in the AAA1 domain of Yta7 (Fig. 1), we were interested to narrow down the region on Yta7 that interacts with Cse4 in the cell. For this purpose, we generated *S. cerevisiae* strains carrying genomically integrated, epitope-tagged N- or C-terminal truncations of Yta7 and tested for coIP with Cse4. The individual truncations were designed based on prior knowledge of the structure of the Yta7 hexamer (Wang et al. 2023). In a first construct (construct A, amino acids (aa) 322–1,379), the N-terminal 321 residues, a relatively unstructured region, were deleted, leaving the bromodomain-interacting motif (BIM) in the protein (Fig. 1a). This Yta7 version was co-IPed with Cse4 and vice versa (Fig. 5, top), showing that the disordered N-terminus was not required for their interaction. Also, the BIM was dispensable for their interaction, because a Yta7 version lacking this region showed co-IP of Yta7 with Cse4 (construct B, aa 418–1,379, Fig. 5, middle). Of note, in the reverse IP of Yta7 construct B to co-IP Cse4, we observed unspecific binding of Yta7 to the beads, and the co-IP of Cse4 was therefore inconclusive (Supplementary Fig. 5a). Furthermore, we observed that sequences C-terminal to the bromodomain (BRD) (construct C, aa 322–1,126) were dispensable for interaction between Cse4 and Yta7, as the respective Yta7 version still showed co-IP with Cse4 (Fig. 5, bottom). Curiously, a Yta7 variant lacking C-terminal sequences beyond BRD, but retaining the complete N-terminus (construct D, aa 1–1,126), was unable to interact with Cse4 in the cell by co-IP, arguing that the N-terminal region has an inhibitory role when the C-terminal domain is absent (Supplementary Fig. 5b). Similarly, a Yta7 construct lacking the BRD and C-terminal sequences also failed to interact with Cse4 (construct E, aa 1–965, Supplementary Fig. 5b). Taken together, these results showed that a core domain of Yta7 containing AAA1 and AAA2, including the BRD, are necessary and sufficient for interaction with Cse4 in the cell.

## Discussion

The proper assembly of CENP-A containing nucleosomes at the centromere is crucial for building a stable kinetochore for chromosome segregation. While CENP-A is deposited in chromatin by dedicated chaperones (HJURP in metazoans, Scm3 in yeast), an important question remains on how this process is regulated by chromatin remodelers. In this work, we identified two mutations in the histone chaperone and chromatin remodeler Yta7 that suppress the defect of *cse4-S135A*. This site in CENP-A^Cse4^ lies close to the DNA of the centromeric nucleosome, and the reduced Cse4-S135A levels at the centromere therefore may result from destabilization of the nucleosome. Importantly, Yta7-R483S restored the levels of Cse4-S135A at the centromere. Furthermore, Yta7-R483S showed a ∼40% increase in catalytic ATPase activity, retained its ability to interact with CENP-A^Cse4^ and to bind to the centromere, and maintained hexamer formation. Together, these results suggest that the hyperactive enzyme directly enhances Yta7's CENP-A^Cse4^ deposition activity. This is noteworthy, because although there are examples of mutations that hyperactivate the catalytic activity in AAA^+^ ATPases such as the K476C mutation in ClpB disaggregase (Oguchi et al. 2012) and A232E mutation in p97 (Niwa et al. 2012), they are exceedingly rare and typically cause a loss of function *in vivo* due to a concurrent loss of other aspects of enzyme function. To our knowledge, the Yta7-R483S is the first example of a mutation within the core AAA^+^ domain that exhibits gain-of-function in both *in vitro* enzymatic activity and *in vivo* function.

In contrast to Yta7-R483S, full-length Yta7-D518E showed decreased rather than increased ATPase activity *in vitro*, but retained interaction with CENP-A^Cse4^ and the centromere as well as structural integrity. Also, Yta7-D518E did not significantly restore Cse4-S135A levels at the centromere. Thus, the mechanism of suppression of this mutant differs from the R483S mutation. D518E is located in the highly conserved Walker B motif. Walker B mutants that inactivate the ATPase activity while maintaining ATP binding, lock the ATPase hexamer in a stable, ATP-bound conformation in many AAA^+^ ATPases, a feature that has been exploited for structural analysis (Puchades et al. 2017; Lo et al. 2019; Wang et al. 2020). The variants used in structural studies of AAA^+^ ATPases are mutations of a conserved glutamate (E) in Walker B to glutamine (Q). The D518E site of Yta7 lies directly N-terminal to this E residue (Fig. 1b), which may explain the reduced ATPase activity of this mutant. Thus, while Yta7-R483S likely activates CENP-A^Cse4^ deposition by directly increasing catalytic activity, Yta7-D518E appears to act through a different yet unknown mechanism, which is also consistent with its weaker suppression phenotype. One possibility is that the D518E mutation impairs ATPase activity, but not ATP binding of Yta7 and thus allows some degree of unfolding of Cse4.

We furthermore found that the interaction of Cse4 with Yta7 in yeast cells requires the AAA1, AAA2 and BRD domains of Yta7, while the disordered N-terminus, including the acidic N-terminal domain (ANR), and the C-terminus beyond BRD are dispensable. Given our current understanding of Yta7 and its homologs, the most likely scenario is that Cse4 (perhaps as a Cse4/H4 dimer or tetramer) interacts with Yta7 on the top of the three-tiered stack, with BRD and AAA1, and that the Cse4 N-terminus inserts into the central cavity of the hexamer.

Altogether, these findings corroborate the notion that Yta7 unfolds Cse4/H4, and that enhanced Yta7 activity by unusual, hypermorphic *yta7* alleles can compensate for defects in Cse4 localization to the centromere. This underscores the importance of chromatin remodeling for the structure and function of centromeric chromatin, which is further highlighted by our finding of *fun30-M385R* as a suppressor of defective Cse4 deposition. Interestingly, M385 lies at the C-terminal end of the SAM-key domain of Fun30 (aa 279–385), a deletion of which causes defects in DNA repair and telomeric silencing akin to *fun30Δ* and abrogates the *in vitro* nucleosome remodeling activity (Karl et al. 2023), though the molecular defect of the allele found here remains to be determined.

Furthermore, the identification of hypermorphic alleles of Yta7 may inform the interpretation of mutations of ATAD2 in cancer cells (Liu et al. 2022). While ATAD2 has been described as a transcriptional regulator of the expression of kinetochore components (CENP-E) (Guruvaiah et al. 2023) and has been found associated with CENP-A in human cells (Remnant et al. 2019), whether it has a more direct role in the assembly or disassembly of centromeric chromatin remains to be determined. Future experiments in this direction will be important to see whether ATAD2 mutation or overexpression contributes to chromosome instability by affecting the functionality of centromeric chromatin.

## Supplementary Material

iyag035_Supplementary_Data

## Data Availability

The authors affirm that all data necessary for confirming the conclusions of the article are present within the article, figures, and tables. Strains and plasmids are available upon request. Supplementary Tables 1 and 2 contain information about the yeast strains and plasmids used. Supplementary Tables 3 and 4 contain information and primer sequences for the construction of truncated versions of *YTA7*. Sequencing reads from WGS were deposited in the National Center for Biotechnology Information (NCBI) Sequence Read Archive (SRA) at http://www.ncbi.nlm.nih.gov/sra under accession no. PRJNA1274834. Supplemental material available at GENETICS online.
