# Supplementary material for: Deposition of CENP-ACse4 is enhanced by mutations in the AAA+ ATPase domain of ATAD2Yta7
Source: Genetics. 2026 Feb 5;232(4):iyag035. doi: 10.1093/genetics/iyag035 (PMC13050212; doi:10.1093/genetics/iyag035)
Supplement: iyag035_Supplementary_Data [file iyag035_supplementary_data.pdf]

Supplementary Tables

Supplementary Table S1: *S. cerevisiae* strains used in this study

Strain *	Genotype
AEY1	*MATα ade2-101 his3-11,15 trp1-1 leu2-3,112 ura3-1* (W303)
AEY4816	*MATα cbf1Δ::NatMX ade2-101 his3-11,15 trp1-1 leu2-3,112 ura3-1*
AEY4846	*MATα cse4Δ::kanMX ade2-101 his3-11,15 trp1-1 leu2-3,112 ura3-1 can1-100 lys2Δ cbf1Δ::NatMX + pRS313-CSE4-3xHA*
AEY5688	*MATa cse4Δ::kanMX ade2-101 his3-11,15 trp1-1 leu2-3,112 ura3-1 can1-100 lys2Δ* + pRS426-*CSE4*-3xHA
AEY6451	*MATa cbf1Δ::NatMX yta7Δ::KanMX ade2 LYS2*, W303
AEY6533	*MATα ade2-101 his3-11,15 trp1-1 leu2-3,112 ura3-1 can1-100 YTA7-9xmyc::KanMX*
AEY6536	*MATα ade2-101 his3-11,15 trp1-1 leu2-3,112 ura3-1 can1-100 YTA7-9xmycKanMX cse4Δ::NatMX* + pRS426-*CSE4*-3xHA
AEY6582	*MATa cbf1Δ::NatMX cse4Δ::KanMX okp1-R164C ade2-101 lys2Δ* (W303) + pRS426-3xHA-*CSE4*
AEY6584	*MATa cbf1Δ::NatMX cse4Δ::KanMX ame1-273* ade2-101 lys2Δ* (W303) + pRS426- 3xHA-*CSE4*
AEY6629	*MATα cse4Δ::KanMX ade2-101 his3-11,15 trp1-1 leu2-3,112 ura3-1 can1-100 lys2Δ cbf1Δ::NatMX* + pRS313-3xHA *cse4-T133A-S135A-E136A*
AEY6630	*MATa cbf1Δ::NatMX cse4Δ::KanMX okp1-R164C ade2-101 lys2Δ* (W303) + pRS313-3xHA-*CSE4*
AEY6631	*MATa cbf1Δ::NatMX cse4Δ::KanMX ame1-273* ade2-101 lys2Δ* (W303) + pRS313- 3xHA-*CSE4*
AEY6632	*MATa cbf1Δ::NatMX cse4Δ::KanMX okp1-R164C ade2-101 lys2Δ* (W303) + pRS313-3xHA *cse4-T133A-S135A-E136A*
AEY6633	*MATa cbf1Δ::NatMX cse4Δ::KanMX ame1-273* ade2-101 lys2Δ* (W303) + pRS313-3xHA *cse4-T133A-S135A-E136A*
AEY6653	*MATα cse4Δ::KanMX cbf1Δ::NatMX yta7Δ::Ura3MX ade2-101 his3-11,15 trp1-1 leu2-3,112 ura3-1 can1-100 lys2Δ* + pRS313-3xHA *cse4-T133A-S135A-E136A*
AEY7277	*MATα ade2-101 his3-11,15 trp1-1 leu2-3,112 ura3-1 can1-100 YTA7 (1-955aa)-9xmyc::hphNT*
AEY7280	*MATα ade2-101 his3-11,15 trp1-1 leu2-3,112 ura3-1 can1-100 YTA7 (1-1126aa)-9xmyc::hphNT*
AEY7286	*MATα ade2-101 his3-11,15 trp1-1 leu2-3,112 ura3-1 can1-100 GALS promotor::natNT2::YTA7(322 – 1379aa)-9xmyc::KanMX*
AEY7299	*MATa cse4Δ::kanMX ade2-101 his3-11,15 trp1-1 leu2-3,112 ura3-1 can1-100 LYS2 Yta7 (1-955aa)-9xmyc::hphNT* + pRS426-*CSE4*-3xHA
AEY7313	*MATa cse4Δ::kanMX ade2-101 his3-11,15 trp1-1 leu2-3,112 ura3-1 can1-100 LYS2 Yta7 (1-1126aa)-9xmyc::hphNT* + pRS426-*CSE4*-3xHA

AEY7329	*MATα ade2-101 his3-11,15 trp1-1 leu2-3,112 ura3-1 can1-100 GALS promotor::natNT2::YTA7 (322 – 1379aa)-9xmyc::KanMX cse4Δ::hphNT* + pRS426-*CSE4*-3xHA
AEY7330	*MATα ade2-101 his3-11,15 trp1-1 leu2-3,112 ura3-1 can1-100 GALS promotor::natNT2::YTA7(418 – 1379aa)-9xmycKanMX*
AEY7362	*MATα ade2-101 his3-11,15 trp1-1 leu2-3,112 ura3-1 can1-100 GALS promotor::natNT2::Yta7(322 – 1126aa)-9xmyc::hphNT*
AEY7370	*MATa ade2-101 his3-11,15 trp1-1 leu2-3,112 ura3-1 can1-100 LYS2 GALS promotor::natNT2::Yta7(322 – 1126aa)-9xmyc::HygromycinB cse4Δ::KanMX* + pRS426-*CSE4*-3xHA
AEY7404	*MATa ade2-101 his3-11,15 trp1-1 leu2-3,112 ura3-1 can1-100 LYS2 GALS promotor::natNT2::YTA7(418 – 1379 aa)-9xmycKanMX cse4Δ::hphNT* + pRS426-*CSE4*-3xHA
AEY7430	*MATα cbf1Δ::NatMX ade2-101 his3-11,15 trp1-1 leu2-3,112 ura3-1 can1-100 yta7-D518E*
AEY7435	*MATα cbf1Δ::NatMX ade2-101 his3-11,15 trp1-1 leu2-3,112 ura3-1 can1-100 yta7-R483S*
AEY7441	*MATα ade2-101 his3-11,15 trp1-1 leu2-3,112 ura3-1 can1-100 Yta7-9xmycKanMX cse4::NatMX* + pRS423-*cse4*-S135A-3xHA
AEY7443	*MATα cse4Δ::kanMX ade2-101 his3-11,15 trp1-1 leu2-3,112 ura3-1 can1-100 lys2Δ cbf1Δ::NatMX* + pRS416-3xHA-*cse4-S135A*
AEY7446	*MATa cse4-S135A::HISMX cbf1Δ::NatMX ade2-101 his3-11,15 trp1-1 leu2-3,112 ura3-1 can1-100*
AEY7449	*MATα cbf1Δ::NATMX cse4-S135A::HISMX yta7-R483S ade2-101 his3-11,15 trp1-1 leu2-3,112 ura3-1 can1-100*
AEY7453	*MATa cbf1Δ::NATMX cse4-S135A::HISMX yta7-D518E ade2-101 his3-11,15 trp1-1 leu2-3,112 ura3-1 can1-100*
AEY7457	*MATα cbf1Δ::NatMX cse4::kanMX ade2-101 his3-11,15 trp1-1 leu2-3,112 ura3-1 can1-100 LYS2 yta7-D518E + pRS426-3xHA-CSE4*
AEY7458	*MATa cbf1Δ::NatMX cse4::kanMX ade2-101 his3-11,15 trp1-1 leu2-3,112 ura3-1 can1-100 LYS2 yta7-R483S + pRS426-3xHA-CSE4*
AEY7469	*MATα cbf1Δ::NatMX cse4::kanMX ade2-101 his3-11,15 trp1-1 leu2-3,112 ura3-1 can1-100 LYS2 yta7-D518E* + pRS416-3xHA-*cse4-S135A*
AEY7470	*MATa cbf1Δ::NatMX cse4Δ::kanMX ade2-101 his3-11,15 trp1-1 leu2-3,112 ura3-1 can1-100 LYS2 yta7-R483S* + pRS416-3xHA-*cse4-S135A*
AEY7482	*MATα cse4Δ::kanMX ade2-101 his3-11,15 trp1-1 leu2-3,112 ura3-1 can1-100 LYS2 yta7-R483S-9xmyc::hphNT1* + pRS423-*cse4*-S135A-3xHA
AEY7476	*MATα cse4::kanMX ade2-101 his3-11,15 trp1-1 leu2-3,112 ura3-1 can1-100 LYS2 yta7-D518E-9xmyc::hphNT1* + pRS423-*cse4*-S135A-3xHA
CY16	*MATa ade2-1 trp1-1 leu2-3, 112 his3-11, 15 ura3-1 can1-100 bar1::HisG lys2;;HisG pep4::unmarked* + pRS303-*YTA7*-TEV-FLAG
CY469	*MATa ade2-1 trp1-1 leu2-3, 112 his3-11, 15 ura3-1 can1-100 bar1::HisG lys2;;HisG pep4::unmarked* + pRS303-*yta7*-R483S-TEV-FLAG

CY470	*MATa ade2-1 trp1-1 leu2-3, 112 his3-11, 15 ura3-1 can1-100 bar1::HisG lys2;;HisG pep4::unmarked* + pRS303-*yta7*-R483S-TEV-FLAG

* Strains were from the laboratory collection or were generated in the course of this study. All AEY strains are isogenic to W303.

Supplementary Table S2: Plasmids used in this study

Plasmid	Description
pAE625	pRS313-3xHA-*CSE4*
pAE977	pRS426-3xHA-*CSE4*
pAE1718	pRS313-3xHA-*cse4-T133A, S135A, E136A*
pAE2886	pRS416-3xHA-*cse4-T133A, S135A, E136A*
pAE3033	pRS416-3xHA-*cse4-T133A*
pAE3034	pRS416-3xHA-*cse4-S135A*
pAE3035	pRS416-3xHA-*cse4-E136A*
CB16	pRS303-*YTA7-TEV-FLAG*
CB469	pRS303-*yta7-R483S-TEV-FLAG*
CB470	pRS303-*yta7-D518E-TEV-FLAG*
CB393	pFastBac1-*YTA7-TEV-FLAG*
CB477	pFastBac1-*D518E-TEV-FLAG*
CB478	pRS303-*yta7-R483S-TEV-FLAG*

Supplementary Table S3: Construction of strains for co-IP of Yta7 truncations with Cse4

Yta7 version	Amino acids	Strain with *YTA7*-9xmyc	Strain with *YTA7*-9xmyc and *CSE4*-3xHA	Primers used for construction of *YTA7* truncation

A	322 - 1379	AEY7286	AEY7329	N-terminal truncation: YTA7-S1 + Yta7_960bp-S4<br>C-terminal tag: Yta7-S3_55bp_fwd + Yta7-S2_55bp_rev
B	418 - 1379	AEY7330	AEY7404	N-terminal truncation: YTA7-S1 + Yta7_1251bp-S4<br>C-terminal tag: Yta7-S3_55bp_fwd + Yta7-S2_55bp_rev
C	322 – 1126	AEY7362	AEY7370	N-terminal truncation: YTA7-S1 + Yta7_960bp-S4<br>C-terminal truncation and tag: Yta7_3375bp-S3 + Yta7-S2_55bp_rev
D	1 - 1126	AEY7280	AEY7313	C-terminal truncation and tag: Yta7_3375bp-S3 + Yta7-S2_55bp_rev + Yta7-S2_55bp_rev
E	1 - 955	AEY7277	AEY7299	C-terminal truncation and tag: Yta7_2862bp-S3 + Yta7-S2_55bp_rev

Supplementary Table S4: Oligonucleotides used in this study

Name	Sequence
Yta7-WT-S4	TGCATCTTCAACGTCGCTACCGCGTCTATTCCTTAAATTTCGTGCC ATCGATGAATTCTCTGTCG
Yta7_960bp-S4	GTCATTGCCACCAAAAGGTCCACCAGTGGGAAAAAGCCTTCTTGTC ATCGATGAATTCTCTGTCG
Yta7_1251bp-S4	GTGGTAAGGCCACCATTTCCTTTAATTGGTCAATGTAGTTGTCTAA CATCGATGAATTCTCTGTCG
Yta7_2862bp-S3	TCAAACGCAGCTCCAACAAATTTTGATGAAAATGGTGAGCCACTGC GTACGCTGCAGGTCGAC
Yta7_3375bp-S3	GCTACTCGCCAAAGGGATTTGGAAAGGCAGGAACTTTTCTTAGAAC GTACGCTGCAGGTCGAC

Yta7-S3_55bp_fwd	ATCAGCATGGGATAAAACAGGAACTGTCGATGAAATAATAAAATTTT TATCTGAACGTACGCTGCAGGTCGAC
Yta7-S2_55bp_rev	TGTATATGAACTAACTACATTTAAGAATTATATAAACATTATGGACTC CTGCTTAATCGATGAATTCGAGCTCG
YTA7-S1	TCTCTCTTCTCGCTATTTATTCACAATTGGTGCAGAAAGAACATGCG TACGCTGCAGGTCGAC
Cen4 up	GCCAGAAATAGTAACTTTTGCCTAAATCAC
Cen4 down	GCTATGAAAGCCTCGGCATTTTGG
POL1 (5‘ fwd	GCTGCAAGCCGCTCGAAA
POL1 (5‘ rev)	CCAGTGTCTTCATCACTT
YTA7 R483S fwd	GTAAGGGAGCTGACATCCTGAGCAAGT
YTA7 R483S rev	TCATGAAGAAAGTGATCTTGCGCTCGTC
YTA7 D518E fwd	GAAATCGACGGACTGGCCCCAGTGCGCT
YTA7 D518E rev	CTCGAAGAAGATGATGGAGGGCTGGTGCTT

Supplementary Figures

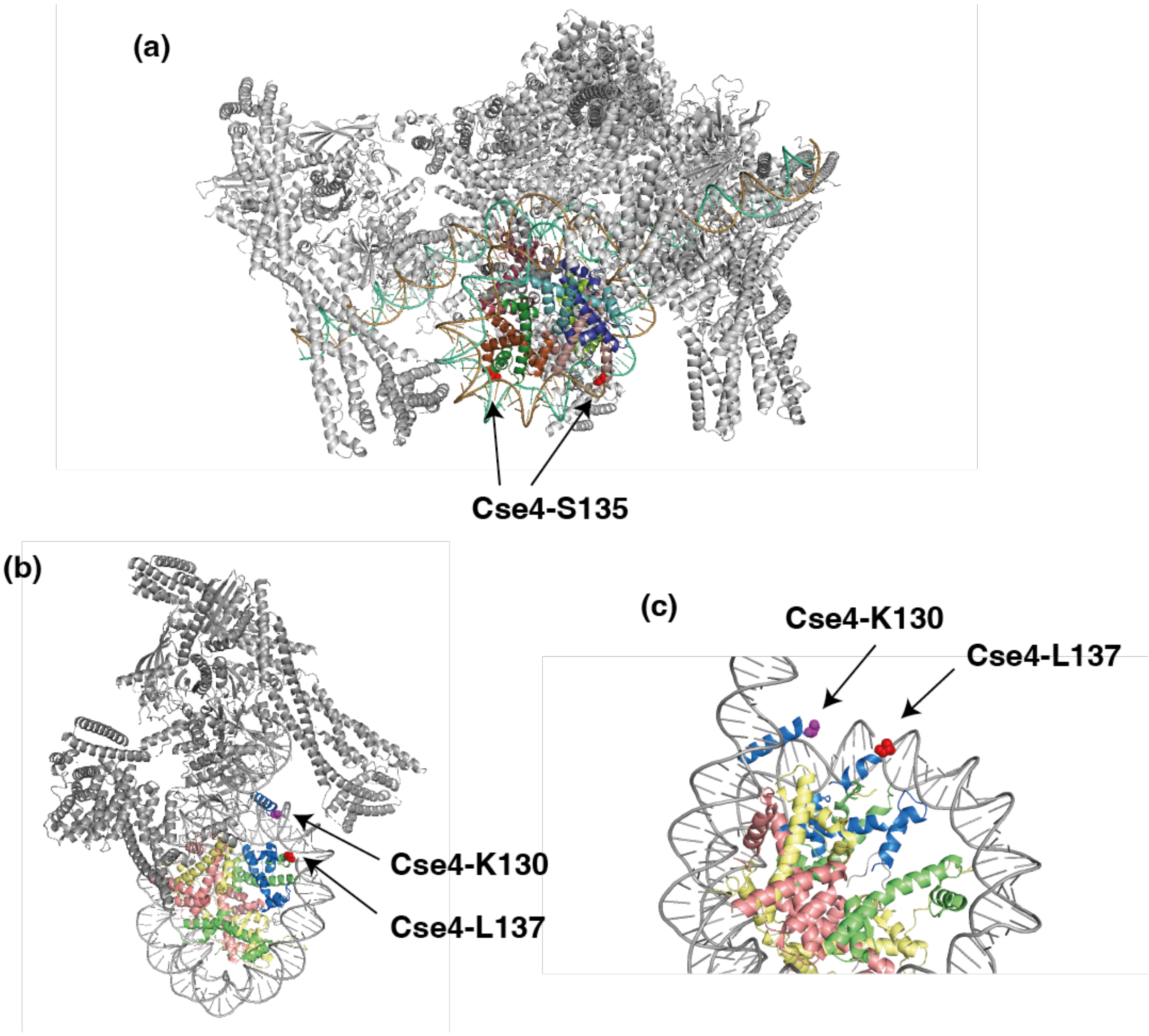

Figure S1: Serine 135 of CENP-A ^{Cse4} lies close to the DNA of the centromeric nucleosome. a) Localization of Cse4 serine 135 in the structure of the yeast inner kinetochore (PDB 8OW1; DENDOOVEN *et al.* 2023). Components other than the nucleosome are colored in grey for clarity. b) and c) Localization of the residues K130 and L137 of Cse4 in the structure of CCAN bound to the Cse4 nucleosome (PDB 6QLD; YAN *et al.* 2019). Cse4-S135 is not resolved in the structure. CCAN components are shown in grey (b) or omitted (c) for clarity.

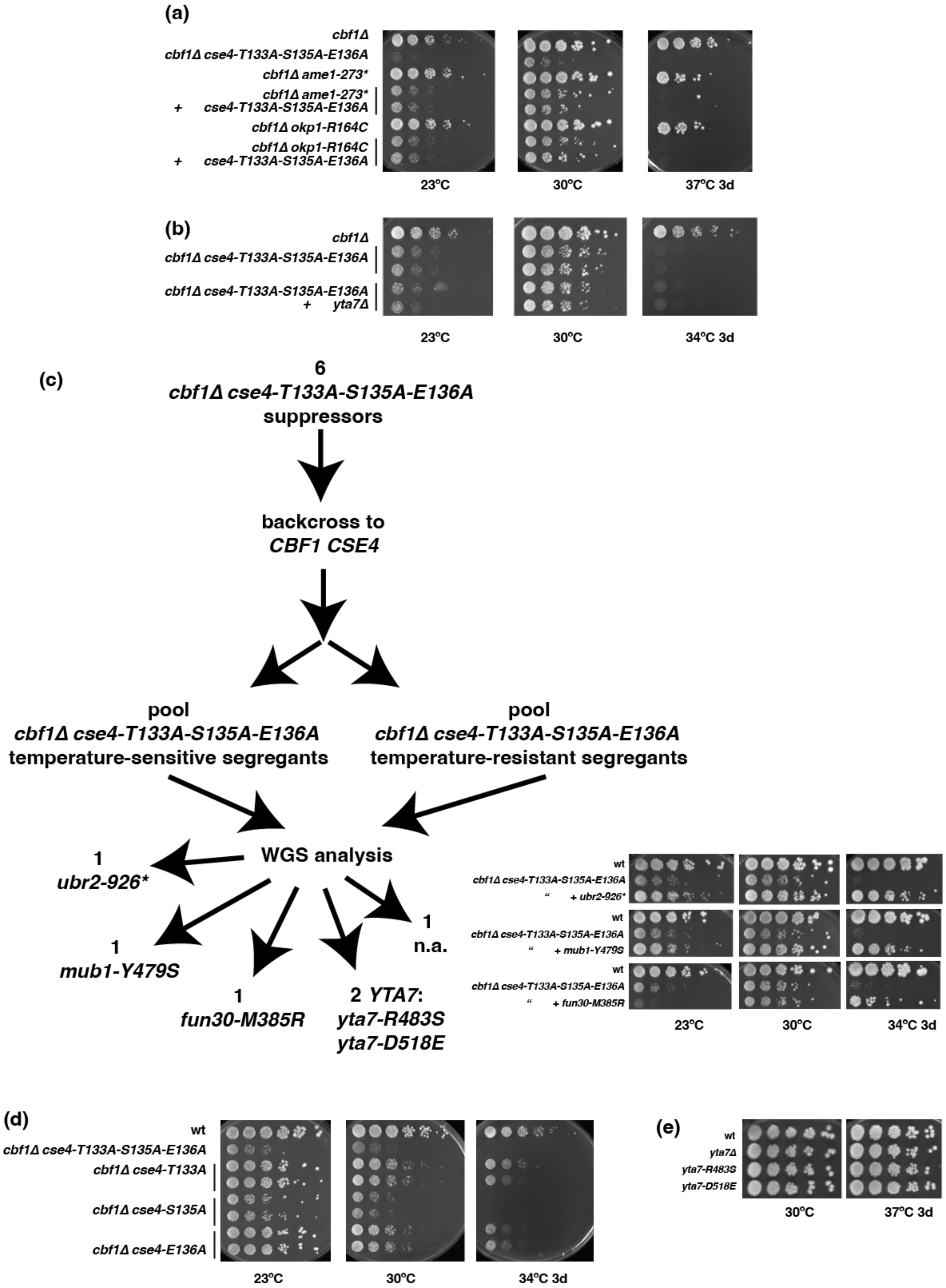

Figure S2: Isolation of suppressors of *cse4-T133A-S135A-E136A cbf1Δ*. a) *cse4-T133A-S135A-E136A* displayed a temperature-sensitive defect in *cbf1Δ*, which was not suppressed by *ame1-273** (a stop codon after amino acid 273 of Ame1) or *okp1-*

R164C. Serial dilutions of the indicated strains were spotted on full medium (YPD) and incubated for 3 days at the different temperatures. b) *yta7Δ* did not suppress the defect of *cse4-T133A-S135A-E136A cbf1Δ*. Representation as in a). c) Left: Flow chart for the isolation of extragenic suppressors of the *cse4-T133A-S135A-E136A cbf1Δ* defect. Six temperature-resistant derivatives (spontaneous or UV-induced) of the *cse4-T133A-S135A-E136A cbf1Δ* strain (AEY6629) were backcrossed, and temperature-resistant and -sensitive segregants were pooled and subjected to whole-genome sequencing, the expectation being that the suppressor mutation is present in the temperature-resistant, but not -sensitive segregants. Whole-genome sequencing (WGS) was performed on the pools, and bioinformatic analysis was performed to identify mutations that are present only in the temperature-resistant segregants. This resulted in the identification of one allele in *UBR2* (*, a stop codon after amino acid 926), *MUB1*, *FUN30* and two alleles of *YTA7*. The causative mutation of a sixth segregant could not be identified (n.a.). The righthand panel shows the growth phenotype of the *ubr2*, *mub1* and *fun30* mutants in *cbf1Δ cse4-T133A-S135A-E136A*. d) *cse4-S135A* is sufficient to cause a temperature-sensitive defect in *cbf1Δ*. e) *yta7-R483S* and *yta7-D518E* do not cause a temperature-sensitive growth defect.

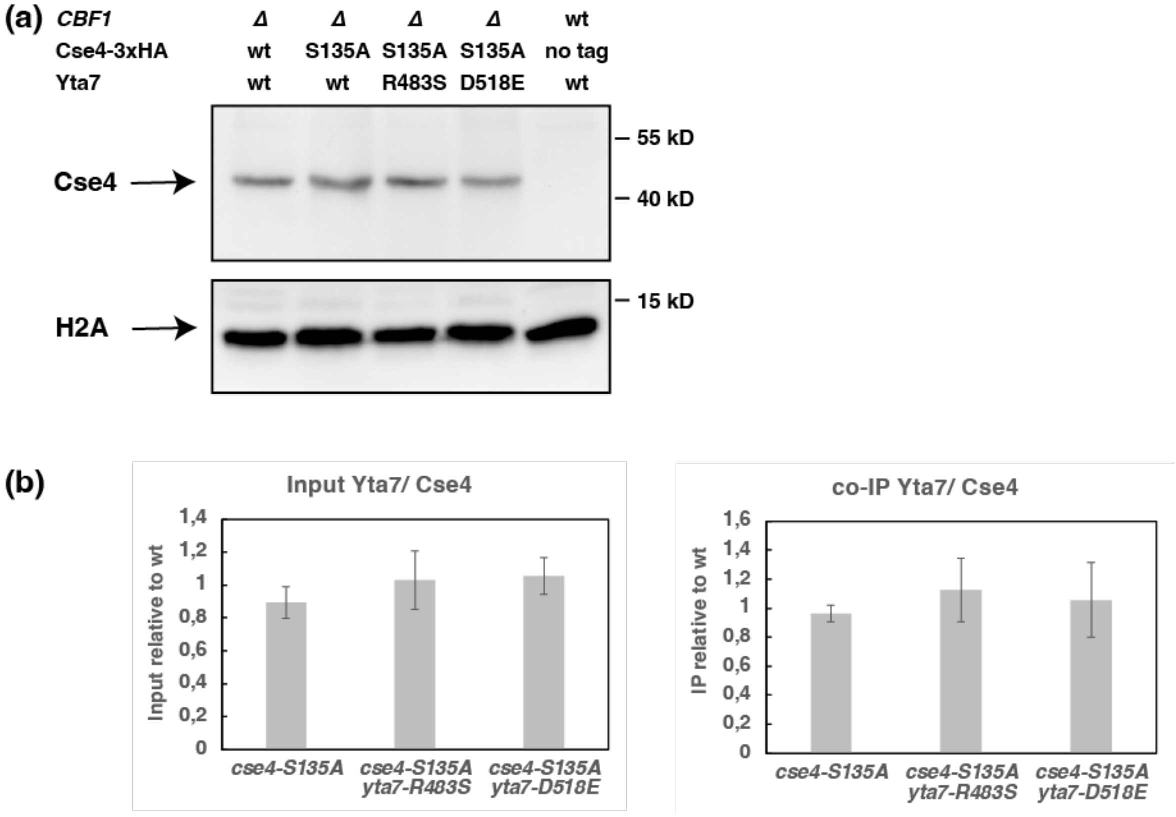

Figure S3: a) The levels of Cse4 protein are unaffected by the *cse4-S135A* and *yta7-R483S/ -D518E* mutations in *cbf1Δ* strains. Total protein extracts of strains with the indicated genotypes were separated by SDS-PAGE, followed by Western blotting and incubation with an α-HA antibody (top) to detect Cse4-3xHA and an α-H2A antibody (bottom, loading control). A strain with untagged Cse4 ("no tag") was used as a control. Molecular weight markers are given on the right. b) Quantification of input levels (left) and co-immunoprecipitation (co-IP, right) of Yta7 and Cse4. Both total protein levels and co-IP were unaffected by the indicated mutations in *CSE4* and *YTA7*. Mean values of four biological replicates with standard deviation are given.

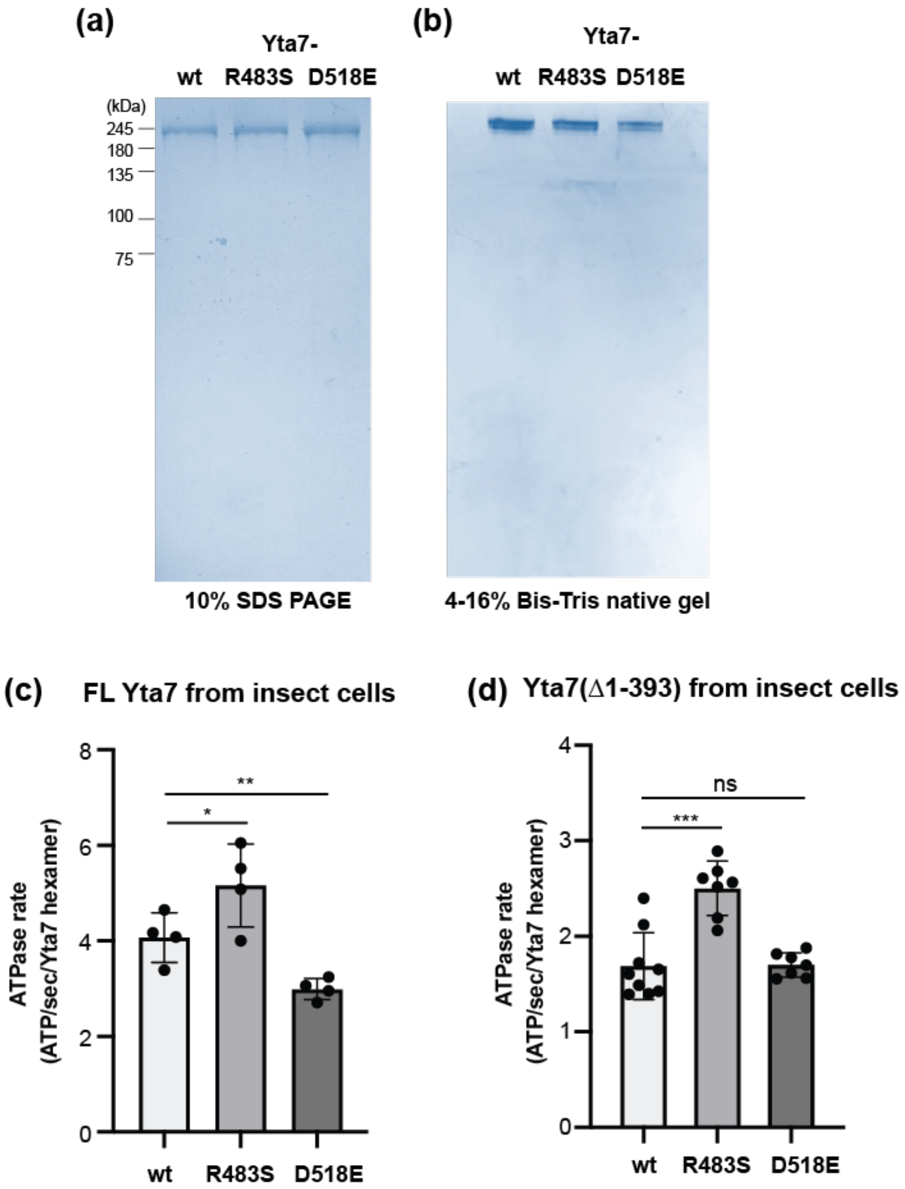

Figure S4: Biochemical characterization of Yta7 mutant proteins purified from *S. cerevisiae* and insect cells. a) 10% SDS-PAGE of Yta7 proteins purified from *S. cerevisiae* showing protein purity. b) 4-16% Bis-Tris native gel of Yta7 proteins purified from *S. cerevisiae* showing the homogeneity of Yta7 proteins. c) Steady state ATPase rates of full-length (FL) Yta7 proteins expressed and purified from insect cells. Mean values for Yta7-wt, -R483S, and D518E are 4.1±0.5, 5.3±0.8, and 3.0±0.2 ATP/sec/Yta7 hexamer, respectively. d) Steady state ATPase rates of N-terminal (aa 1-393) truncated Yta7 proteins expressed and purified from insect cells. Mean values for Yta7-wt, -R483S, and D518E are 1.7±0.4, 2.5±0.3, and 1.7±0.1

ATP/sec/Yta7 hexamer, respectively. Error bars represent SD, *** $p<0.001$, ** $p<0.01$, * $p<0.1$.

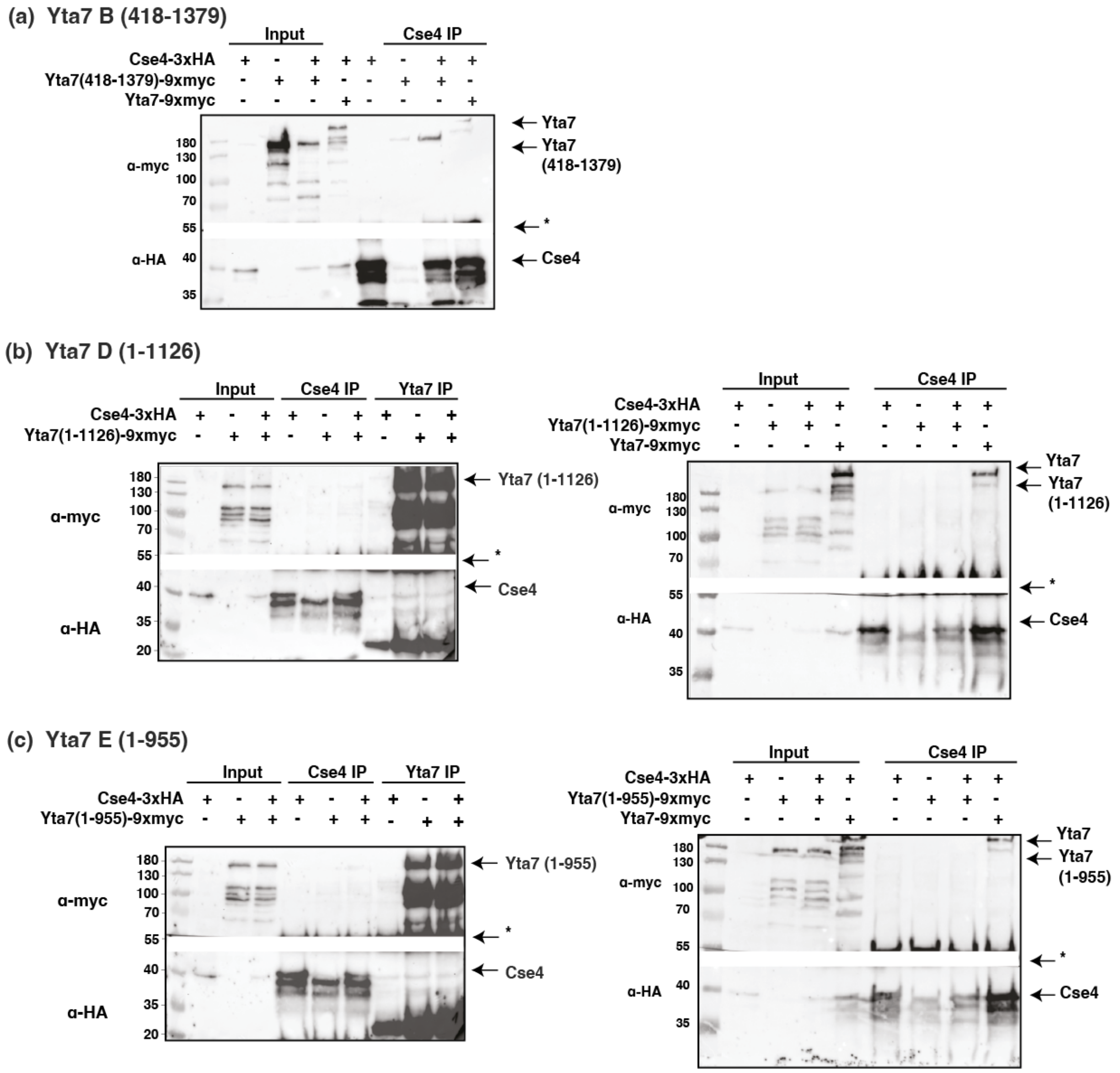

Figure S5: Yta7 (aa 1 – 1126) and (aa 1 – 955) did not show *in vivo* interaction with Cse4, and Yta7 (aa 418 – 1379) interacted nonspecifically with the beads. The Yta7 constructs B, D and E (see Figure 1) were epitope tagged with 9xmyc in yeast strains carrying Cse4-3xHA. Whole-cell extracts (input) were precipitated with Cse4 or Yta7, and the presence of Yta7 and Cse4 was determined by Western blotting, as shown in Figure 3 (upper part of blot developed for Yta7 detection, lower part of the same blot for Cse4). a) Precipitation with α-HA (Cse4-IP) showed unspecific enrichment of Yta7 in the absence of tagged Cse4. b) No co-IP of Yta7 construct D and Cse4. c) No co-

IP of Yta7 construct E with Cse4. The asterisk (*) indicates the place where the blot was cut (white box). See also Figure 5.

References

Dendooven, T., Z. Zhang, J. Yang, S. H. McLaughlin, J. Schwab *et al.*, 2023 Cryo-EM structure of the complete inner kinetochore of the budding yeast point centromere. Sci Adv 9**:** eadg7480.

Yan, K., J. Yang, Z. Zhang, S. H. McLaughlin, L. Chang *et al.*, 2019 Structure of the inner kinetochore CCAN complex assembled onto a centromeric nucleosome. Nature 574**:** 278-282.
